# Optimization of metabolism to improve efficacy during CAR-T cell manufacturing

**DOI:** 10.1186/s12967-021-03165-x

**Published:** 2021-12-07

**Authors:** Meng Zhang, Xin Jin, Rui Sun, Xia Xiong, Jiaxi Wang, Danni Xie, MingFeng Zhao

**Affiliations:** 1 First Center Clinical College, Tianjin Medical University, Tianjin, 300192 China; 2 Department of Hematology, Tianjin First Central Hospital, Tianjin, 300192 China; 3 School of Medicine, Nankai University, Tianjin, 300071 China

**Keywords:** Metabolism, CAR-T, Immunotherapy, Glycolysis, OXPHOS

## Abstract

Chimeric antigen receptor T cell (CAR-T cell) therapy is a relatively new, effective, and rapidly evolving therapeutic for adoptive immunotherapies. Although it has achieved remarkable effect in hematological malignancies, there are some problems that remain to be resolved. For example, there are high recurrence rates and poor efficacy in solid tumors. In this review, we first briefly describe the metabolic re-editing of T cells and the changes in metabolism during the preparation of CAR-T cells. Furthermore, we summarize the latest developments and newest strategies to improve the metabolic adaptability and antitumor activity of CAR-T cells in vitro and in vivo.

## Background

CAR-T cell therapy is the fourth most common tumor treatment option after surgery, chemotherapy, and targeted therapy, and is successful in the treatment of malignant hematological tumors. CAR recognizes tumor-associated antigen targets and activates intracellular signals that stimulate proliferation of T cells, which identify and kill tumor cells. The complete remission rate (CR) in the treatment of recurrent/refractory B-cell acute lymphoblastic leukemia and B-cell lymphoma (R/R B-ALL)with CAR-T cell therapy is more than 90% and 50%, respectively [1, 2]. However, CAR-T cell therapy includes disadvantages of high recurrence rate and poor effect in solid tumors [3–6]. A key reason for this is dysregulated tumor cell metabolism, which confers large amounts of metabolic interference to CAR-T cells, resulting in functional failure of CAR-T cell therapy. Therefore, there is an urgent need to identify novel strategies for enhancing CAR-T cell therapy.

**Fig. 1:**
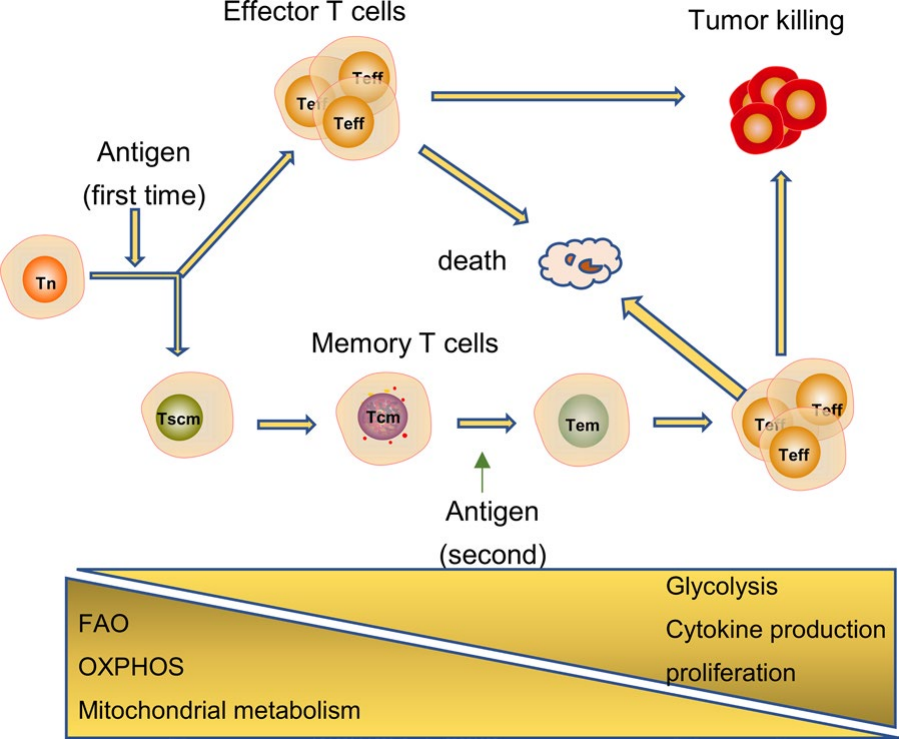
T cells have different metabolic requirements according to their function. The metabolism of Tn cells mainly depends on OXPHOS and FAO to provide energy. After encountering the antigen, Tn cells become activated and differentiat into Teff cells. During this process, glycolysis is enhanced, while mitochondrial metabolism and OXPHOS are weakened. When the antigen is cleared, most Teffs undergo apoptosis and a small number of them differentiate into memory T cells. At this time, metabolism must be converted from glycolysis to FAO

Traditional CAR-T manufacturing method involves six steps: (1) getting peripheral blood mononuclear cells (PBMCs); (2) Enriching T cells; (3) t cell activation; (4) transduction; (5) CAR-T cell expansion; (6) patient infusion. This traditional process typically takes 2 to 6 weeks. The new method can reduce the autologous CAR-T cell manufacturing time from an industry norm of 2 to 6 weeks. FasT CAR platform are able to concurrently activate and transduce resting T cells into a single “concurrent activation-transduction” step using XLenti vectors derived from lentivirus. An abstract from the ASH annual meeting shows that during CAR-T cell manufacturing each stage has different functional and metabolic requirements and small changes can have large effects on the efficacy and side effects of CAR-T cell therapy [7] (Fig. 1).

Recently, a number of studies have reported approaches for optimizing the metabolism of CAR-T cells at different stages to improve their antitumor activity. In vitro expansion of T cells and CAR-T cells requires proper metabolism to maintain a undifferentiated state, which must extend through the in vivo use to enhance antitumor activity. However, in vivo, it becomes necessary to enhance metabolic activity to maintain high invasion potential and cytotoxic function. In this review, we first briefly describe the metabolic re-editing of T cells and changes in metabolism during CAR-T cell preparation. Additionally, we summarize the latest developments and newest strategies for improving the metabolic adaptability and antitumor activity of CAR-T cells in vitro and in vivo.

## T cell metabolic reprogramming

T cell subgroups are divided into naive T cells (Tn), effector T cells (Teff), central memory (Tcm), effect memory (Tem), and others [8], each with different functional and metabolic requirements. T cell activation and metabolic reprogramming occur at the same time. The metabolism of Tn mainly depends on oxidative phosphorylation (OXPHOS) and fatty acid oxidation (FAO) to provide energy. After encountering the antigen, the TCR and CD28 synergize to activate PI3K-AKT-mTOR pathway. T cell activation signaling pathways (e.g., the PI3K-AKT-mTOR pathway) activate transcription factors (e.g., HIF-1α, c-Myc), which upregulate glucose transporter protein type 1 (GLUT1) expression to promote glycolysis [9, 10]. However, whether it is activated by cross-linking the TCR/CD3 complex or connected to CD28 alone, it will not cause a significant change in Glut1 expression [10]. Together, this allows the cell to meet metabolic needs for rapid proliferation and cytokine production. And this lead to Tn cells differentiate into Teff cells. The metabolic pattern of memory T cells is similar to that of Tn, but with a slightly higher degree of OXPHOS and mitochondrial spare respiratory capacity, which allows memory T cells to quickly activate upon encountering an antigen [11]. Once the antigen is cleared, most Teffs undergo apoptosis and a small number differentiate into memory T cells. At this time, metabolism must to be converted from glycolysis to FAO [12]. Metabolism is a major driving factor that determines the fate of memory T cells [13, 14]. Activated T cells regulate metabolism to control the number and types of metabolic intermediates, which are used to control the epigenetic response of key gene transcription and promote differentiation of T cells into memory T cells [15, 16]. However, the specific mechanism involved in this process remains poorly understood.

Most currently available CAR-T therapies are manufactured using lentiviruses. When manufacturing CAR-T cells, we first obtain PBMCs through leukapheresis or Ficoll. PBMCs are purified to obtain T cells, which are then stimulated with anti-cluster of differentiation 3 (CD3)/CD28 magnetic beads to promote proliferation and differentiation. At this time, the metabolism is reprogrammed (from FAO to glycolysis). During the activation process, cells that require more glucose tend to differentiate into Teffs, whereas those that require less glucose preferentially form memory T cells [17]. Next, T cells are transduced, most commonly via lentivirus infection. That is, the obtained T cell subsets are incubated with the lentiviral vector encoding CAR. Studies have shown that the composition of T cell subsets and their metabolic adaptability are closely related to their antitumor activity. CD19 CAR-T cells that were manufactured from purified CD4+ or CD8+ Tcm or Tn have been shown to have enhanced metabolic adaptability and long-term anti-tumor response [18, 19]. CD19 CART derived from TSCM showed good long-term response as compared to CD19 CART standardly manufactured, however it is unclear whether just having more Tscm or the decreased glycolysis of the Tscm drives the long-term response [20]. And research has shown that this is likely related to early memory phenotype and FAO-dependent OXPHOS [21]. Finally, CAR-T cells are expanded to a certain number in vitro. After resuspension and purification, cells are returned to the patient via infusion. Clinical studies have shown that 19 CAR-T cells, which have a higher ratio of memory T cells and glycolysis, increase the chance for CR in chronic lymphocytic leukemia cell (CLL) patients [22]. Other conditions in the process of manufacturing CAR-T, such as infection temperature, can also affect the differentiation of CAR-T. Whether it affects metabolism needs further study via lentiviral technology [23].

Sleeping Beauty and PiggyBac transposition are nonvirus-based technologies that produce a higher ratio of central memory T cells [24, 25](2016 and 2017 by The American Society of Hematology). 2020 ASH Meeting announced the safety and efficacy of its potential first-in-class GC012F FasT CAR-enabled dual-targeting BCMA/CD19 cell therapy in patients with relapsed or refractory multiple myeloma (Unpublished data). How CAR-T cells metabolism changes during this process and the influence of different methods on CAR-T metabolism needs further research.

## Strategy for optimizing metabolism to improve efficacy during CAR-T cells production

**Fig. 2:**
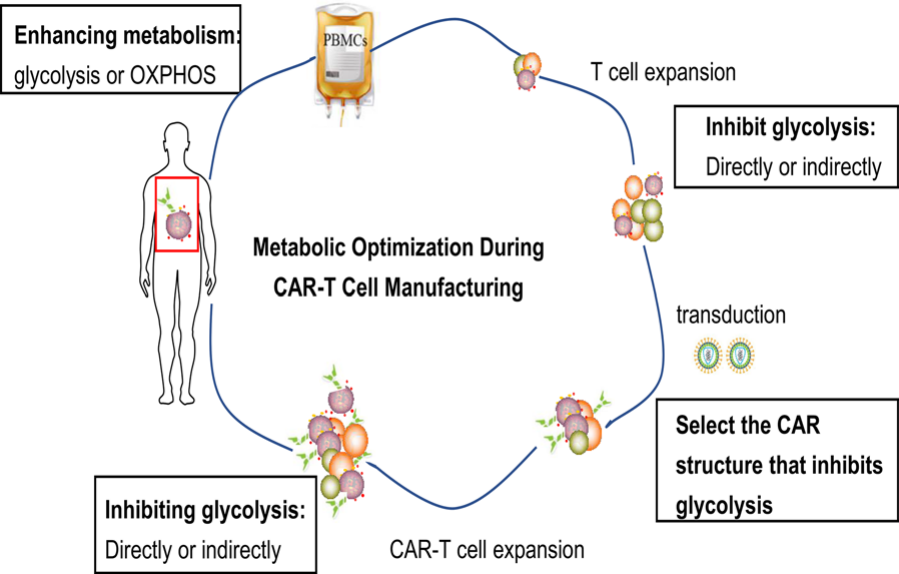
In vitro expansion of T cells and CAR-T cells require proper metabolism(directly or indirectly inhibit glycolysis) to maintain undifferentiated state, which can extend the duration in vivo and enhance anti-tumor activity; However, in vivo it is necessary to enhance metabolic (glycolysis or OXPHOS) activity to maintain high invasion potential and killing function

The differentiation and metabolic state of CAR-T cells, as well as T cells that are used to manufacture CAR-T cells, play an important role in regulating antitumor activity. Since the CAR-T cell manufacturing process is easily manipulated, T cell differentiation can be altered by optimizing metabolism to improve antitumor activity. Each stage of the CAR-T cell manufacturing process requires a different type of metabolism. During the in vitro growth and manufacturing stage of CAR-T cells, T cells are transduced with a CAR and grown to large numbers prior to patient infusion. Appropriate inhibition of metabolism can maintain the undifferentiated state and improve antitumor activity. However, CAR-T cells require robust metabolic activity in vivo (simultaneously enhanced glycolysis and mitochondrial metabolism) to support the production of biosynthetic intermediates for cell proliferation (Fig. 2).

### Inhibiting glycolysis during T cell expansion to limit differentiation

As mentioned above, once obtained, purified T cells must be expanded in vitro for 3 days before being used to manufacture CAR-T cells. During this process, cytokines are added regularly in addition to CD3/CD28 magnetic beads. Interleukin 2 (IL-2) is the most frequently added cytokine because it promotes glycolysis and rapid proliferation of T cells. IL-2 also mediates the physiological coupling of T cell expansion and effector differentiation; however, the coupling of cell expansion and effector differentiation poses a major therapeutic obstacle to improving the efficacy of immunotherapy [26]. Multiple studies have shown that T cell metabolism is a key regulator of expansion and differentiation [27]. T cell proliferation and differentiation can be uncoupled through manipulation of metabolic pathways. Prior to manufacturing of CAR-T cells, T cells can be maintained in an undifferentiated state by inhibiting glycolysis directly (glycolysis inhibitors inhibit the PI3K/AKT signaling pathway) or indirectly (promoting mitochondrial function, optimizing medium).

#### Direct inhibition of glycolysis to optimize T cell function

A synthetic glucose analogue, 2-Deoxy-D-glucose (2DG), has been the most widely studied inhibitor of glucose metabolism since 1950s. The 2DG molecule is transferred to the cytoplasm through Gluts and is phosphorylated by hexokinase to form 2DG-6-phosphate. The phosphorylated form cannot be further metabolized, thereby inhibiting hexokinase. Glycolysis and OXPHOS are partially destroyed by 2DG because it interferes with the first key step of glucose metabolism [28]. Sukumar M found that graded levels of glycolysis can act as a metabolic rheostat determining the decision between memory and terminal effector differentiation in CD8+ T cells. The addition of 2DG during expansion in vitro inhibited T cell glycolysis and promoted formation of memory T cells whether in both tumor and chronic viral. In addition 2-DG could sustain Foxo1 activity and enhances lymphoid homing. In contrast, overexpression of glycolytic enzyme pgam1 impaired T cell implantation and the ability of CD8+ T cells in vivo. Taken together, these data show that high glycolytic metabolism affects the formation of memory T cells [17].

#### Indirect inhibition of glycolysis

The PI3K/AKT signaling pathway is at the core of T cell activation, and it acts downstream of the T cell activation signal [29, 30]; its main function is to promote T cell glycolysis, and it plays a key role in regulating T cell differentiation and activity [31, 32]. However, continuous activation of this pathway promotes terminal differentiation of T cells and loss of memory, which affects T cell function [33]. As such, this pathway acts to regulate the balance between terminal T cell differentiation and memory cell production. The addition of PI3K inhibitors, AKT inhibitors, or mTOR inhibitors during the expansion of T cells has the potential to reduce glycolytic activity, increase the percentage of Tn and Tcm, and promote the killing function of CD8+ T cells and adoptive cell therapy [34–36]. This may be related to inhibition of transcription factors that promote memory, such as forehead box transcription factors (FOXO) and TCR/lymphoid enhancer factor (LEF)/β-catenin, as well as enhancement of signal transducer and activator of transcription 3 (STAT3) activity in human T cells. This may also be related to increased expression of genes that encode several of the transcriptional regulators involved in the generation and maintenance of long-lived memory cells, including transcription factor, FOXO1, B cell lymphoma 6, STAT3, and TCR/LEF/β-catenin [37–39].

It is worth noting that the above studies are all inhibiting the effect of CD8+ T cell glycolysis on its differentiation. Similar to CD8+ T cells, AKT-inhibition (AktiVIII) preserve memory differentiation of CD4+ Tn cells and promotes CD4+ Th-subset skewing toward Th2-associated cells. However, in the presence of CD4+ T cells, the favorable effect of AKT-inhibition on the functionality of CD8+ T cells drastically diminished [40]. Furthermore the PI3K/AKT signaling pathway likely affect multiple signaling pathways not all related to glycolysis. Whether PI3K and AKT inhibitors affect T cell differentiation through other ways remains to be further studied.

Mitochondria are dynamic organelles that are involved in cell proliferation, migration, metabolism, and death [41], and are essential for immunotherapy. Promoting mitochondrial function is a key metabolic approach that promotes the generation of memory T cells [42]. Studies have shown that mitochondrial membrane potential (ΔΨm), mitochondrial dynamics, and mitophagy reflect the function of mitochondria and are related to the differentiation and function of T cells. CD8+ T cells with low or high ΔΨm can be distinguished based on absorption of the lipophilic cationic dye, tetramethyl rhodamine methyl ester. Low ΔΨm T cells have enhanced metabolic adaptability and the metabolic characteristics of memory CD8+ T cells [43]. Isolation of T cells based on ΔΨm is a new metabolic method that is helpful to screen for the best T cell subsets for CAR-T cell manufacturing.

Several studies have linked mitochondrial dynamics (fission/fusion) to immunotherapy. The morphology of mitochondria is closely related to cell metabolism and differentiation [44, 45]; for example, mitochondria of Tn cells are small and fragmented, and basic energy consumption is maintained by OXPHOS [46]. Teff cells have more linear, punctate mitochondria as a result of mitochondrial fission while Tm cells maintain fused networks; and Teff cells also have reduced levels of OXPHOS and increased levels of aerobic glycolysis [45, 47]. In memory CD8+ T cells, mitochondrial fusion and mass increase, which is manifested as a slender mitochondrial network [47]. Inhibition of mitochondrial fission or promotion of fusion may increase OXPHOS levels as well as the killing function of T cells. For instance, CD8+ T cell memory generation is promoted by the mitochondrial fission inhibitor Mdivi-1, inhibition of Drp1 (the key medium of mitochondrial fission), and overexpression of OPA1 (the key medium to promote integration) [45, 47, 48].

Mitophagy is an important regulatory mechanism for maintaining the quality and integrity of mitochondria. Mitophagy is thought to play a selective role in CD8+ T cell effector memory formation. However, the regulatory mechanism of mitophagy during T cell central memory formation requires experimental verification in future studies.

In addition, T cells lacking serine/threonine Pim kinase or pretreatment of T cells with the Pim kinase inhibitor AZD1208 significantly reduced glycolytic metabolism and promoted tumor clearance efficiency of melanoma-specific pmel T cells [49]. In summary, during the in vitro t cell activation stage, various metabolic pathways can be used to maintain T cells in a low differentiated state. If this approach is used prior to making CAR-T cells, it could improve CAR-T cells function.

### CAR-T cell preparation stage: selecting the CAR structure that inhibits glycolysis to maintain low differentiation

The structure of CAR contains a single chain fragment of variable region antibody, a transmembrane domain, a costimulatory molecule, and an intracellular signal domain. CAR-T cell technology has now been developed in five generations. The products currently on the market are all second-generation CAR-T cells. In the CAR-T cell preparation stage, the CAR structure that inhibits glycolysis can be used to maintain naive and improve the efficacy of CAR-T cell therapy. For example, selecting costimulatory factors that favor OXPHOS and co-expression of the PI3K inhibitory region that inhibits glycolysis.

#### Selecting costimulatory factors that favor OXPHOS

The costimulatory/co-suppressive signal not only regulates survival, proliferation, and differentiation of T cells, but also the metabolic pathways [50]. For example, CD28 costimulatory factor can promote T cell glycolysis by up-regulating GLUT1, pyruvate dehydrogenase kinase 1 (PDK1), or by activating mTOR [10, 51–53]. Unlike CD28, CD137 (41-BB) appears to be a mitochondrial stimulant; it promotes FAO mainly through the LKB1-AMP-activated protein kinase (AMPK) signaling pathway. Additionally, the expression of peroxisome proliferator-activated receptor γ coactivator 1-α (PGC1α, a protein that promotes mitochondrial biogenesis) and OPA-1 can be controlled to enhance mitochondrial biogenesis and dynamics [54–56]. Other stimulating factors such as OX40 can increase GLUT1 expression and enrich T cells with genes involved in glycolysis and fatty acid synthesis [56, 57]. Inducible costimulatory (ICOS) promotes glucose uptake and metabolism via the mTOR signaling pathway [58].

Compared with the first generation of CAR-T cells, the second generation fused the intracellular part of costimulatory factors (such as CD28 and 41-BB) upstream of the CD3 domain, which significantly enhanced the killing effect. A further understanding of the downstream pathways of costimulatory factor signaling will help select CAR intracellular sequences and improve the antitumor effects of CAR-T cells. Previous reports have shown that CD28 CAR-T cells consume glucose relatively quickly when CD28 CAR-T and 41-BB CAR-T cells were stimulated, and there were more effector memory T cells in CAR-T cell subtypes [59]. Similar to T cells, GLUT1 and one of the key glycolytic enzymes PDK1 were increased in the CAR-T group containing CD28, and extracellular acidification was higher. This suggests that the CD28 based CAR-T cells favor glycolysis [59],which is consistent with previous research in T cells. The phosphorylation of CAR CD3ζ, Lck, ZAP-70, and LAT increases after encountering a tumor cell [60]. Early phosphorylation promotes Ca [2]^+^ influx, CD69 expression, and secretion of cytokines (IL-2 and IFN-γ), which makes CAR-T cells proliferate rapidly and kill tumor cells [61]. However, this may lead to early failure, poor persistence, and high incidence of cytokine release syndrome (CRS) [62].

41-BBz ζ CAR T cells maintained higher levels of SRC, mitochondrial biogenesis, and increase OXPHOS in comparison to 28z CAR T cells [59]. When encountering tumor cells, 41BB CAR-T cells proliferate slowly but persistently, showing favorable metabolic characteristics and the CAR-T cell subtype (Tcm). In addition, Clinical trials have shown that patients treated with 4-1BBζ CD19 CAR-T had better MRD- CR rates and longer 1 year EFS as compared to CD28 ζCD19 CAR-T, this may be associated with a significantly higher peak expansion of 4-1BBζ CAR-T cells than that seen in the CD28ζ CAR-T cell group [63, 64]. These data once again demonstrate that proper inhibition of glucose metabolism in vitro contributes to the antitumor effect of CAR-T cells. For CAR-T cells that include ICOS and OX40 [65, 66], co-stimulation is not the same with respect to subtype, function, cytokine secretion, or persistence. The in vitro metabolism of CAR-T cells with different co-stimulants requires further examination to help select the best CAR structure.

#### Co-expression of the PI3K inhibitory region inhibits glycolysis

The CAR structure of CRB-402 (bb21217) BCMA CAR-T contains a PI3K inhibitory region. A phase I clinical trial showed that 46 R/R multiple myeloma had a median follow-up of 8.5 months, 18% ≥ CR, 30% VGPR, 67% developed CRS, 3% ≥ grade 3, and 22% developed CAR-related encephalopathy syndrome. Although the effect of this CAR structure and other structures has not been evaluated, the result of PBMC analysis shows more enriched memory-like T cells (LEF1^+^, CD27^+^, CCR7^+^), which have previously proved to be related to CAR-T cells persistence and killing function. However, it remains to be determined specifically how the PI3K/AKT/mTOR signaling pathway uniquely regulates glucose metabolism between T cell subpopulations or in different states [67].

### Expansion of CAR-T cells in vitro: inhibiting glycolysis and maintaining an undifferentiated state

CAR-CD3ζ ITAM signaling in CAR-T cells can promote differentiation compared to T cells, making CAR-T cells more likely to differentiate into Teff and Tem cells with a result of poorer CAR-T cell persistence in vivo [68]. Therefore, it is particularly important to uncouple CAR-T cell proliferation and differentiation by regulating metabolic pathways during CAR-T cell expansion of.

#### Direct inhibition of glycolysis

These findings suggest that by optimizing metabolism, T cells can be maintained in an undifferentiated state. Similar to the activation of T cells in vitro, in the process of expanding CAR-T cells in vitro, we should appropriately weaken glycolysis so that CAR-T cells are more likely to differentiate into Tn and Tcm cells. These cells types improve antitumor activity and have a prolonged persistence in vivo. For example, through the use of glycolysis inhibitors 2-DG or inhibition of the PI3K/AKT signaling pathway, appropriate inhibition of glycolysis helps to maintain stem-like [68–71]. Nevertheless, inhibition of glycolysis or the PI3K/AKT/mTOR signaling pathways may inhibit CAR-T cell proliferation while also affecting differentiation [68]. Hence, the selective PI3Kδ inhibitor Idelalisib (CAL-101) [36, 72] and the selective PI3Kβ (p110β) isoform inhibitor GSK2636771 [73] may reduce the impact on the absolute CAR-T cells count.

#### Indirect inhibition of glycolysis by promoting mitochondrial function

Mitochondrial plasticity is closely related to T cell differentiation, and it has been shown that mitochondrial biomass can be used to assess metabolic adaptation of CAR-T cells [74]. In addition, mitochondria may be a key hub in regulating CAR-T cell differentiation, persistence, and failure. During in vitro expansion, CAR-T cell metabolism can be shifted to OXPHOS by adjusting mitochondrial membrane potential, fusion/fission, and autophagy. This would ultimately promote differentiation into CAR-T memory cells and improve killing function. However, no studies have shown the role of mitochondrial biosynthesis in regulating metabolic adaptability and function of CAR-T cells during the CAR-T cell preparation.

### Optimizing media

Generally CAR-T cells are produced and expanded in vitro using a medium that is rich in nutrients like carbohydrates and amino acids [75]. However, the lack of oxygen and nutrients in the tumor microenvironment (TME) limits the expansion and function of CAR-T cells. Therefore, in the process of amplification, optimizing the composition of the medium and appropriately inhibiting the glycolysis of CAR-T cells serves to improve the metabolic adaptability and maintain a low differentiation state. For example, the addition of arginine can promote OXPHOS and inhibit glycolysis [76]. The addition of carnosine can neutralize the extracellular protons produced by aerobic glycolysis and can shift CAR-T cell metabolism from glycolysis to OXPHOS [75].

In particular, IL-2, the most common cytokine added to CAR-T cells culture, promotes glycolysis for rapid T cell proliferation. However, it may also drive terminal differentiation or activation-induced cell death [77]. Adjusting the concentration and timing of IL-2 addition to culture media can reduce CAR-T cell side effects and increase efficacy (increasing the proportion of memory CAR-T cell subsets). Other types of cytokines, such as IL-15, improve metabolic adaptation and preserve the stem cell memory phenotype of CAR-T cells by reducing mTORC1 activity and inhibiting their glycolytic activity [78]. IL-21 shifts metabolism towards FAO and OXPHOS to promote the formation of Tcm cells [79]. IL-7 and IL-15 can not only induce the formation of Tscm, but also reverse CAR-T cell depletion [80]. In summary, these data demonstrate that appropriate optimization of metabolism can produce CAR-T cells with strong metabolic adaptability. In order to make CAR-T cells reach the best state before infusion, further research is needed on the ratio of the best medium components (including cytokines, nutrients, etc.).

In short, CAR-T cells can maintain a less-differentiated state directly through inhibition of glycolysis or indirectly via enhancing mitochondrial metabolism. This adjustability of metabolism provides opportunities to promote CAR-T cell function.

### Strategies to promote CAR-T cells function after infusion into the patient

When CAR-T cells are infused into the patient, the in vivo metabolism need must be considered, and it is opposite that found in vitro. T cells with high metabolic activity can better kill tumor cells [81]. Nevertheless, the metabolism of CAR-T cells is affected by hypoxia and nutritional deficiencies. CLLs reduce the glucose uptake of T cells and impair mitochondrial biogenesis [82]. Recent studies have shown that enhancing either glycolysis or mitochondrial metabolism in vivo can improve the function of CAR-T cells. In addition, due to the competitive metabolism between tumor cells and CAR-T cells, it can also promote the function of CAR-T cells in vivo by inhibiting the metabolism of tumor cells.

## Enhancing glucose metabolism

In the TME, transforming growth factor-β (TGF-β) affects T cell glycolysis and OXPHOS, which further affects T cell function. Inhibiting TGF-β through CRISPR can promote the long-term therapeutic effect of CAR-T cells on solid tumors [83, 84]. An abstract from the ASCO annual meeting in 2019 (an clinical study)show that CART-PSMA-TGFβRdn, which co-expresses dominant negative TGF-β, is safe and effective in the treatment of prostate cancer [85]. A reversible CRS has been observed that is responsive to tocilizumab. Inhibiting TGF-β may simultaneously increase glucose metabolism and OXPHOS of CAR-T cells in the TME. However, further data is needed to support this.

In addition, the inhibitory cytokines and molecules in TME activate phosphatase and counteract the AKT signal [86]. In the ALL mouse model, the expression of GLUT1 or AKT increases T cell effector function [87]. In order to overcome the down-regulation of AKT expression in TME, enabling CAR-T cell overexpression of Akt improves antitumor efficacy [88]. T cells co-transduced WITH caAkt-GD2-CAR produce more Th1 cytokines and granzyme B, which increased cytotoxic activity against LAN-1 neuroblastoma cells [89]. However, continuous Akt activation drives terminal differentiation and loss of CD8+ T cell memory [33]. It is proposed above that inhibiting the PI3K/AKT signaling pathway during CAR-T cell culture in vitro can delay CAR-T cell differentiation by inhibiting glycolysis, making the phenotype tend to last longer memory T cells. However, CAR-T cells that overexpress AKT enhance the effector function of T cells. These two studies manipulated CAR-T cells from different perspectives to enhance antitumor efficacy and warrant further investigation to determine which approach is superior.

In the TME, tumor cells rapidly consume extracellular glucose, inhibiting T cell glycolysis and the production of the downstream metabolite phosphoenolpyruvate (PEP). In addition to its function in glycolysis, PEP regulates cytoplasmic Ca^2+^ concentration and NFAT1 activation in T cells, and is a key metabolite in regulating the antitumor response. T cells overexpressing PCK1 increase PEP production, limit tumor growth, and prolong survival in melanoma mice [90, 91]. Furthermore mutations or knockouts of Von Hippel Lindau genes [92, 93] or prolyl hydroxylase can enhance glycolysis, promote antitumor activity, and prolong the persistence of T cells. This view seems to contradict the inhibition of glycolysis to extend the persistence of T cells, which once again proves the complexity of T cell metabolism.

## Enhanced mitochondrial metabolism

Research shows that enhanced mitochondrial biogenesis (MB) during CAR-T cell therapy can improve prognosis of CLL patients [82]. PGC-1α is a key regulator of MB and regulates OXPHOS and FAO (the main metabolic characteristics of memory T cells) [94]. Activators of PGC-1α act synergistically with checkpoint inhibitors to increase ROS production in T cells by enhancing mitochondrial metabolic activity, thereby promoting the antitumor effects of T cells [95, 96]. CD8+ TIL-overexpressing PGC1α can improve the quality and function of mitochondria, and restore the antitumor ability of depleted T cells [97, 98]. Recent studies show that CAR-T cells overexpressing PGC1α (mf CAR-T) increased the expression of metabolically-adapted target genes (ERRα, TFAM and NRF2). By improving the quality and function of mitochondria, high tumor killing ability remains under low glucose conditions. mf CAR-T is a promising new strategy to improve the function of CAR-T cells in TME. A preliminary report presented at the 2020 ASH Annual meeting suggests that enhancing the mitochondrial metabolism of immune cells at the tumor site can promote tumor killing [99]. Overexpression of the FOXM1 gene induced CAR-i Tscm and also enhanced antitumor effects by promoting mitochondrial biogenesis, fatty acid synthesis, and OXPHOS [100].

## Inhibition of tumor cell metabolism

Metabolic of tumor cells regulate the metabolic state of the TME. The increased level of glycolysis in tumors is called the "Warburg effect", and the effector function of T cells also depends on glycolysis [101]. A recent publication suggests that glucose is most utilized by myeloid cells in the tumor followed by T cells and tumor cells [102]. Since myeloid cells are the main consumer of nutrients such as glucose, immune and tumor cells are in direct competition for the remaining nutrients in the TME. The glycolytic activity of tumor cells may limit the uptake of glucose by CAR-T cells. Glucose deprivation will not only affect the differentiation of Tn into Teff [32], but also inhibit calcium signal transduction, affecting the production of IFN-γ and the function of cytotoxic T cells [103]. Thus, metabolic competition between tumor cells and T cells may be associated with reduced antitumor function of CAR-T cells, poor curative efficacy, and tumor progression [103–106]. The PD-L1/PD1 axis affects both tumor and T cell metabolism [103]. PD-L1 is expressed on cancer cells that activate Akt-mTOR to promote glycolysis, increase glucose uptake, and enhance glucose competition with T cells. Several studies have demonstrated that blocking the PD-L1/PD1 axis through monoclonal antibodies or an engineered CAR structure improved the efficacy of CAR-T cells [107–109]. One of the reasons is that blocking the PD-L1/PD1 axis directly inhibits glycolysis of tumor cells, restores glucose in the TME, thereby promoting the glycolysis of CAR-T cells and the production of IFN-γ [103]. Another reason is that it may directly promote CAR-T cell metabolism and reverse CAR-T failure [110].

In addition to inhibiting PD-L1/PD1 axis, glycolysis inhibitors can also be used to directly inhibit tumor cell metabolism, however, the metabolic similarities between tumor cells and immune cells may lead to direct inhibition of tumor cell metabolism, which could impact the efficacy of immunotherapy. Selection of GLUT1 inhibitors or ketogenic diets may be potential approaches to inhibit tumor cells glucose metabolism without compromising CAR-T cells function [28].

Some complex technologies such as CRISPR may be used to genetically modify patient T cell metabolism in a clinical setting. CRISPR/Cas9 can knock out diacylglycerol kinase to increase the TCR signal and promote the killing function of CAR-T cells [111]. DGK KO augments TCR distal signaling (ERK phosphorylation was amplified and lasted longer) and thereby increases the effector functions of 139 CAR-T cells [111]. Browsing the CRISPR/Cas9 library and screening results revealed that REGNASE-1 can promote metabolic failure, and Regnase-1-null CAR-T cells show a stronger therapeutic effect than wild-type cells [112].

## Conclusion

Improving CAR-T cell function by regulating metabolism is challenging. Enhancing glycolytic metabolism can promote early proliferation and the production of cytokines (e.g., IFN-γ), but the persistence is poor. Increasing glycolysis of T cells makes them more differentiated into Teff, which can produce more IFN-γ. These cells have high invasive potential, but have a short life and are not conducive to differentiation into memory T cells based on OXPHOS metabolism. On the other hand, the shift towards OXPHOS promotes differentiation into memory T cells, but proliferation may be limited and migratory capacity and killing function may be reduced. In the process of killing tumors, we will need CAR-T cells with a long life, high invasion potential, and high killing function. This prompted us to plan how to better regulate the metabolism of CAR-T cells during the manufacturing process. Properly inhibiting glycolysis (directly or indirectly) during expansion of T cells or CAR-T cells in vitro contributes to memory T cell formation, the latter being directly related to CAR-T cells persistence and therapeutic efficacy in vivo. However CAR-T cell metabolism in vivo needs to be enhanced to better perform the killing function. Recent studies have cloned the γ-subunit of AMPK into a lentiviral vector to increase AMPK signaling in CAR-T cells, which can promote expansion in vitro while promoting the differentiation of memory T cells [113]. A polyethylene glycosylated IL-10 (AM0010) could enhance the cytotoxicity of CAR-T cells both in vitro and in vivo [114]. By genetic modification to a subunit of human IL-2 (ortho-hIL-2), compared with recombinant IL-2, in addition to promoting the proliferation of T cells in vitro, it can also promote T cell proliferation in vivo. A slight adjustment to ortho-hIL-2 through a genetic modification can not only promote T cell proliferation in vitro, but also promote T cell proliferation in vivo [115]. With the development of CRISPR and other disciplines, it may be possible in the future to regulate CAR-T cell metabolism more finely in vivo.

## Data Availability

Not applicable.

## Abbreviations

CAR-T cell: Chimeric antigen receptor T cell
CR: Complete remission rate
R/R B-ALL: Recurrent/refractory B-cell acute lymphoblastic leukemia and B-cell lymphoma
PBMCs: Peripheral blood mononuclear cells
Tn: Naive T cells
Teff: Effector T cells
Tcm: Central memory
Tem: Effect memory
OXPHOS: Oxidative phosphorylation
FAO: Fatty acid oxidation
TCR: T cell receptor
GLUT1: Glucose transporter protein type 1
Tscm: Memory stem T cells
CLL: Chronic lymphocytic leukemia cell
IL-2: Interleukin 2
2DG: 2-Deoxy-D-glucose
FOXO: Forehead box transcription factors
LEF: TCR/lymphoid enhancer factor
STAT3: Signal transducer and activator of transcription 3
PDK1: Pyruvate dehydrogenase kinase 1
AMPK: AMP-activated protein kinase
ICOS: Inducible costimulatory
CRS: Cytokine release syndrome
TME: Tumor microenvironment
PGC-1α: Peroxisome proliferator-activated receptor γ coactivator 1-α
MB: Mitochondrial biogenesis
